# High-fat diet and chronic stress aggravate adrenal function abnormality induced by prenatal caffeine exposure in male offspring rats

**DOI:** 10.1038/s41598-017-14881-0

**Published:** 2017-11-01

**Authors:** Zheng He, Feng Lv, Yufeng Ding, Hegui Huang, Lian Liu, Chunyan Zhu, Youyin Lei, Li Zhang, Cai Si, Hui Wang

**Affiliations:** 1 Department of Pharmacology, Basic Medical School of Wuhan University, Wuhan, 430071 China; 2 Hubei Provincial Key Laboratory of Developmentally Originated Disease, Wuhan, 430071 China; 3 Department of Pharmacy, Tongji Hospital, Tongji Medical College, Huazhong University of Science and Technology, Wuhan, 430030 China

## Abstract

We previously demonstrated thatprenatal caffeine exposure (PCE) suppressed fetal adrenal steroidogenesis and resulted in developmental programming changes in offspring rats. However, whether these changes play a role in adrenal corticosterone synthesis under high-fat diet (HFD) and unpredictable chronic stress (UCS) remains unknown. In present study, rat model was established by PCE (120 mg/kg.d), and male offspring were provided normal diet or HFD after weaning. At postnatal week 21, several rats fed HFD were exposed to UCS for 3 weeks and sacrificed. The results showed that compared with the corresponding control group, the serum corticosterone levels and adrenal steroid synthetase expression of the PCE offspring without UCS were reduced. Moreover, the glucocorticoid (GC)-activation system was inhibited, and insulin-like growth factor 1 (IGF1) signaling pathway expression was increased. With UCS exposure in the PCE offspring, serum corticosterone levels and adrenal steroid synthetase expression were increased, the activity of GC-activation system was enhanced, and adrenal IGF1 signaling pathway expression was decreased. Based on these findings, PCE induced adrenal hypersensitivity in adult male offspring rats, as shown by the reduced corticosterone levels under HFD conditions but significantly enhanced corticosterone levels with UCS, in which GC-IGF1 axis programming alteration may play an important role.

## Introduction

Caffeine is a methylxanthine alkaloid widely found in coffee, tea, soft drinks, food and certain analgesic drugs^1–4^. Worldwide, 50% of all adults consume coffee, and in the US, the average daily caffeine intake during pregnancy is 110 mg/d^5,6^. Epidemiological and animal studies have shown that prenatal caffeine exposure (PCE) has adverse effects on reproductive and embryo toxicity, such as intrauterine growth retardation (IUGR)^7,8^. Another report found that the incidence of adult metabolic syndrome (MS) is 2.53-fold higher in IUGR-affected infants than in those not affected by IUGR^9^. Our previous studies demonstrated that PCE in rodents could lead to overexposure to maternal glucocorticoids (GCs) in IUGR-affected rodent fetuses and hypothalamic-pituitary-adrenal (HPA) axis-associated neuroendocrine metabolic programming alteration. The fetuses showed low basal activity of the HPA axis, enhanced sensitivity of the HPA axis to chronic stress, GC-dependent alterations of blood glucose and lipid, and an increased risk of MS after birth^10–13^.

The adrenal gland is the earliest and fastest developing organ of the HPA axis, which is responsible for the synthesis of GC^14,15^. GC have key roles in altering fetal tissue morphology and function, and steroidogenic acute regulatory protein (StAR) and cholesterol side-chain lyase (CYP11A1) are the key rate-limiting enzymes in steroid hormone synthesis^16,17^. A previous report showed that 11β-hydroxysteroid dehydrogenase (11βHSDs) is distributed widely in the adrenal gland and other tissues in the body. Moreover, 11βHSD can effectively control the active GC concentration in local tissue (represented by cortisol in humans and corticosterone in rodents) by catalyzing reciprocal transformation between inactive and active GCs^18^. Corticoid receptors (CRs), including the mineralocorticoid receptor (MR) and glucocorticoid receptor (GR), are ligand-dependent nuclear transcription factors^19^. The insulin-like growth factor 1 (IGF1) signaling pathway is the core of the endocrine regulatory system, modulates adrenal cell proliferation, differentiation and metabolism, which play important roles in morphology and functional development^20,21^. Our recent studies have found that PCE could cause adrenal function abnormity in offspring rats, which was related to low- expression of adrenal steroid synthetase (the first type of programming)^12,22^ and GC-IGF1 axis programming (the second type of programming) induced by fetal overexposure to maternal GCs^23^.

Many environmental factors (such as high nutritional status and social or mental pressure) can induce and aggravate MS and related metabolic diseases. Given the role of high nutritional status, a high-fat diet (HFD) is a common cause of MS and related metabolic diseases in adults. Research has shown that an HFD is associated with nearly all MS phenotypes, such as hypertension, insulin resistance, diabetes and obesity^24,25^. An HFD can also significantly increase the damage caused by GCs, such as induction of type 2 diabetes^26^. IUGR-affected offspring are more likely to develop metabolic diseases when receiving an HFD^27^. Furthermore, chronic stress due to high social pressure was shown to be another common cause of MS and related metabolic diseases in adults^28,29^. Chronic stress can induce and aggravate the risk of metabolic diseases and mental disorders^30,31^. A recent study demonstrated that reduced birthweight or stress in pregnancy alter mRNA levels of steroid synthetase in male but not female offspring. These effects were associated with metabolic dysfunction^32^. Our previous study demonstrated that serum glucose and lipid levels showed GC-dependent changes in adult offspring rats with PCE under HFD conditions, which led to increased susceptibility to MS in a gender-specific manner^33^. However, whether the adrenal steroid synthesis of male offspring with PCE is changed with HFD or HFD/chronic stress conditions after birth, and the underlying mechanisms mediating the development of abnormal blood corticosterone levels are unclear.

The present study sought to explore the adrenal changes in IUGR-affected male offspring rats caused by PCE by detecting alterations in the steroidal synthetase system, IGF1 signaling pathway and GC-activation system (11βHSDs/CR levels) of the adrenal axis with normal diet, HFD and HFD/chronic stress conditions after birth. Furthermore, based on the two types of intrauterine programming of adrenal developmental toxicity, we elucidated the mechanisms of adrenal functional change, and below, we discuss its significance.

## Results

### Serum corticosterone levels

**Figure 1:**
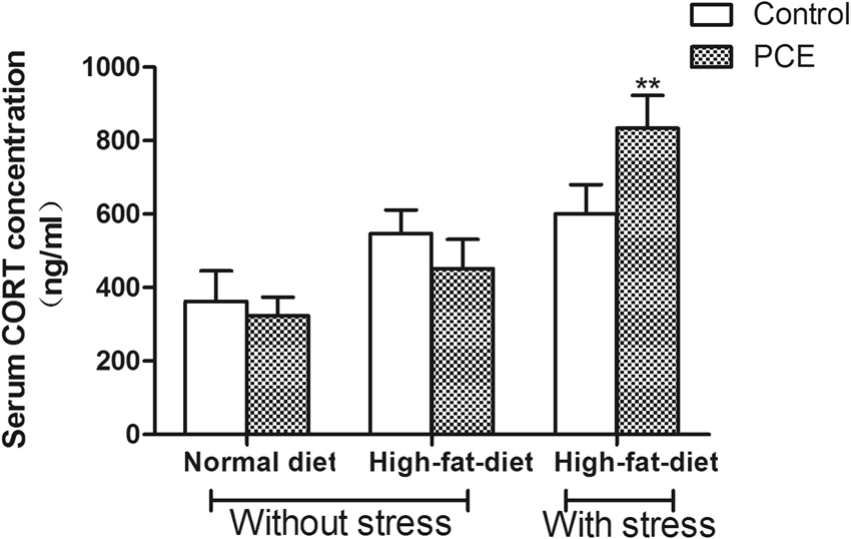
Effects of prenatal caffeine (PCE, 120 mg/kg.d) exposure on serum corticosterone (CORT) with normal and high-fat diets without and with unpredictable chronic stress (UCS) in adult male rat offspring. Mean ± S.E.M., n = 10. ^**^ *P* < 0.01 *vs*. respective control.

Using ELISA technique, we determined serum corticosterone concentrations in adult male offspring in the normal diet, HFD and HFD/chronic stress groups. As shown in Fig. 1, compared with their respective control groups, the serum corticosterone concentrations in the PCE groups showed decreases with a normal diet or HFD and increases (*P* < 0.01) with UCS exposure under HFD conditions.

### Adrenal steroid synthesis function

**Figure 2:**
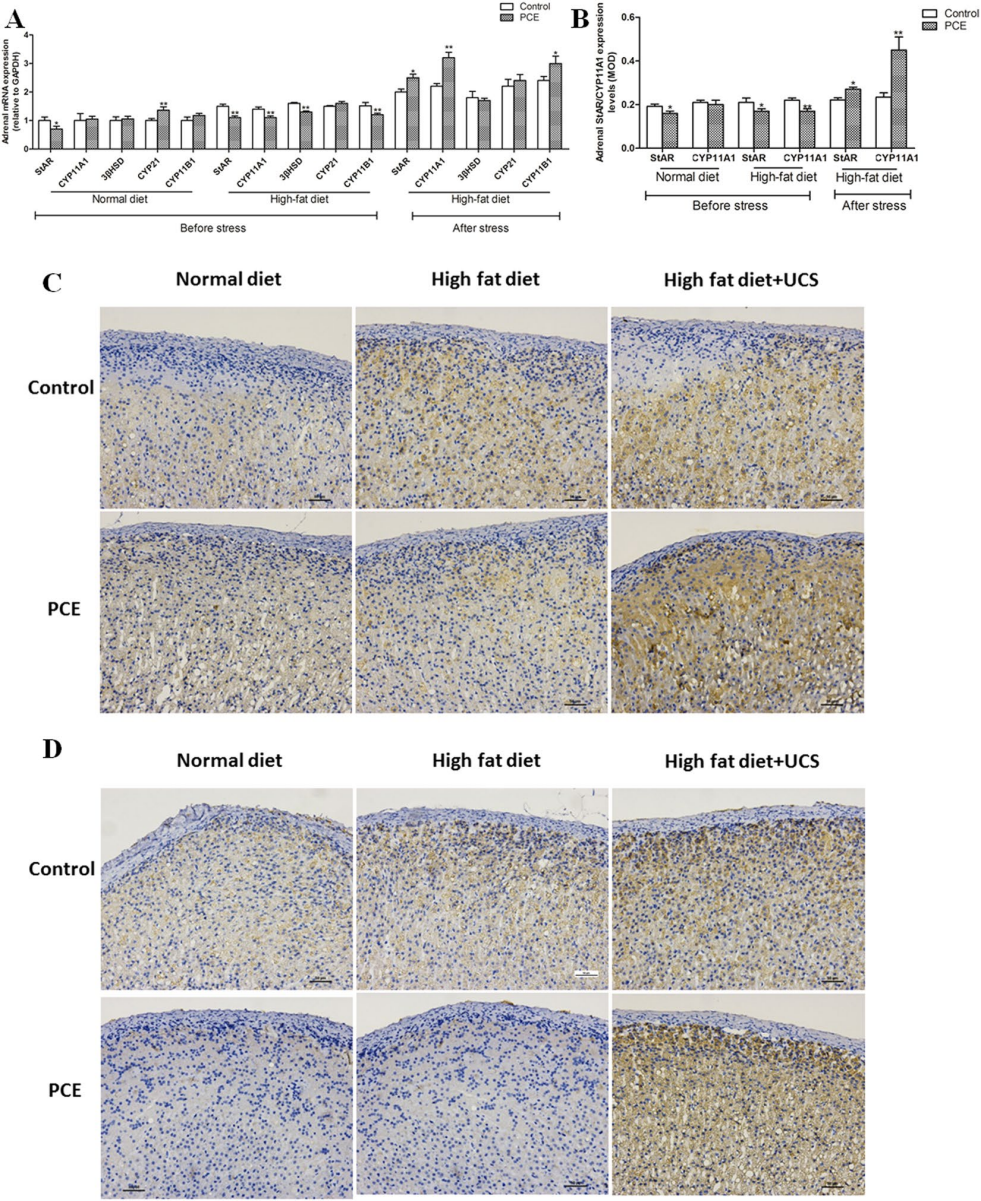
Effects of prenatal caffeine (PCE, 120 mg/kg.d) exposure on the mRNA and protein expression of adrenal steroidogenic enzymes with normal and high-fat diets without and with unpredictable chronic stress in adult male rat offspring. Mean ± S.E.M., n = 5 (the adrenal samples from two litters were counted as one sample for adrenal mRNA). ^*^ *P* < 0.05, ^**^ *P* < 0.01 vs respective control. (**A**) The mRNA expression of adrenal steroidogenic enzymes; (**B**) The mean optical density (MOD) of steroidogenic acute regulatory (StAR) or cytochrome P450 cholesterol side chain cleavage (CYP11A1) in the adrenal cortex; (**C**) The protein expression of StAR (Immumohistochemical staining, ×200); (**D**) The protein expression of CYP11A1 (Immumohistochemical staining, ×200). 3β-HSD: 3β-hydroxysteroid dehydrogenase; CYP21: steroid 21-hydroxylase; CYP11B1: steroid 11β-hydroxylase.

To determine whether the changes in serum corticosterone levels were due to the adrenal steroid synthetase system, we tested the relevant indexes. As shown in Fig. 2A, compared with the respective control, the mRNA expression levels of StAR were decreased and of CYP21 were increased in the PCE group fed a normal diet (*P* < 0.05, *P* < 0.01). Adrenal steroidal synthetase levels in the PCE group were significantly reduced or presented a decreasing trend under HFD condition, including the levels of StAR, CYP11A1, 3β-HSD and CYP11B1 (*P* < 0.01). With HFD/UCS exposure, the adrenal steroid synthetase levels in the PCE group were significantly enhanced, including the levels of StAR, CYP11A1 and CYP11B1 (*P* < 0.05, *P* < 0.01). Adrenal immunohistochemical analyses are shown in Fig. 2B–D. Compared with their respective controls, the protein expression levels of StAR and CYP11A1 were decreased in the PCE groups under normal diet and HFD conditions (*P* < 0.05) and were increased with UCS (*P* < 0.05, *P* < 0.01). These results suggested that the low adrenal steroid synthetase expression continued into adulthood under HFD conditions and was enhanced with UCS exposure in offspring with IUGR induced by PCE.

### Adrenal IGF1 signaling pathway

**Figure 3:**
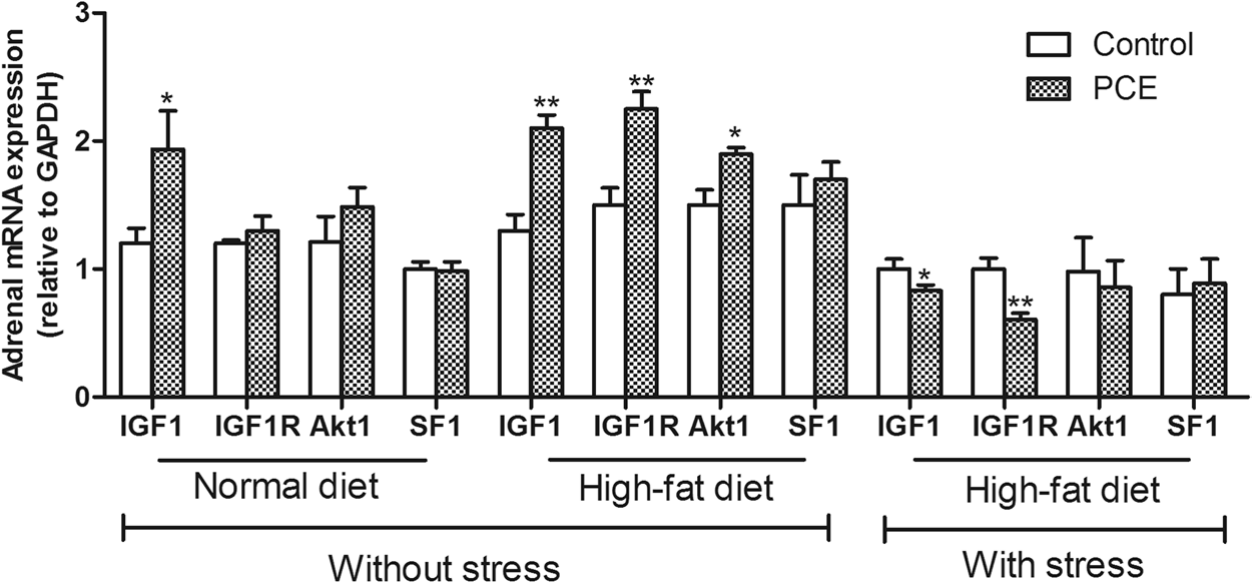
Effects of prenatal caffeine exposure (PCE, 120 mg/kg.d) exposure on the mRNA expression of adrenal insulin-like growth factors 1 (IGF1) pathway with normal and high-fat diets without and with unpredictable chronic stress in adult male rat offspring. Mean ± S.E.M., n = 5, the adrenal samples from two litters were counted as one sample. ^*^ *P* < 0.05, ^**^ *P* < 0.01*vs* respective control. IGF-1R: insulin-like growth factor-1 receptor; AKT1: protein kinase B; SF1: steroidogenic factor 1.

As shown in Fig. 3, compared with the respective control, only the mRNA expression level of IGF1 was increased in the PCE group fed a normal diet (*P* < 0.05). The mRNA levels of all the IGF1 signal pathway components were increased or showed an increasing trend under HFD conditions, such as the levels for IGF1, IGF1R, AKT1 and steroidogenic factor 1 (SF1) (*P* < 0.05, *P* < 0.01). With UCS exposure, the mRNA levels of IGF1 and IGF1R were decreased compared with the levels in the controls (*P* < 0.05, *P* < 0.01). These results suggested that the expression of the adrenal IGF1 signal pathway was enhanced without UCS exposure but decreased with UCS in male offspring with IUGR induced by PCE.

### Adrenal 11βHSD/CR system expression

**Figure 4:**
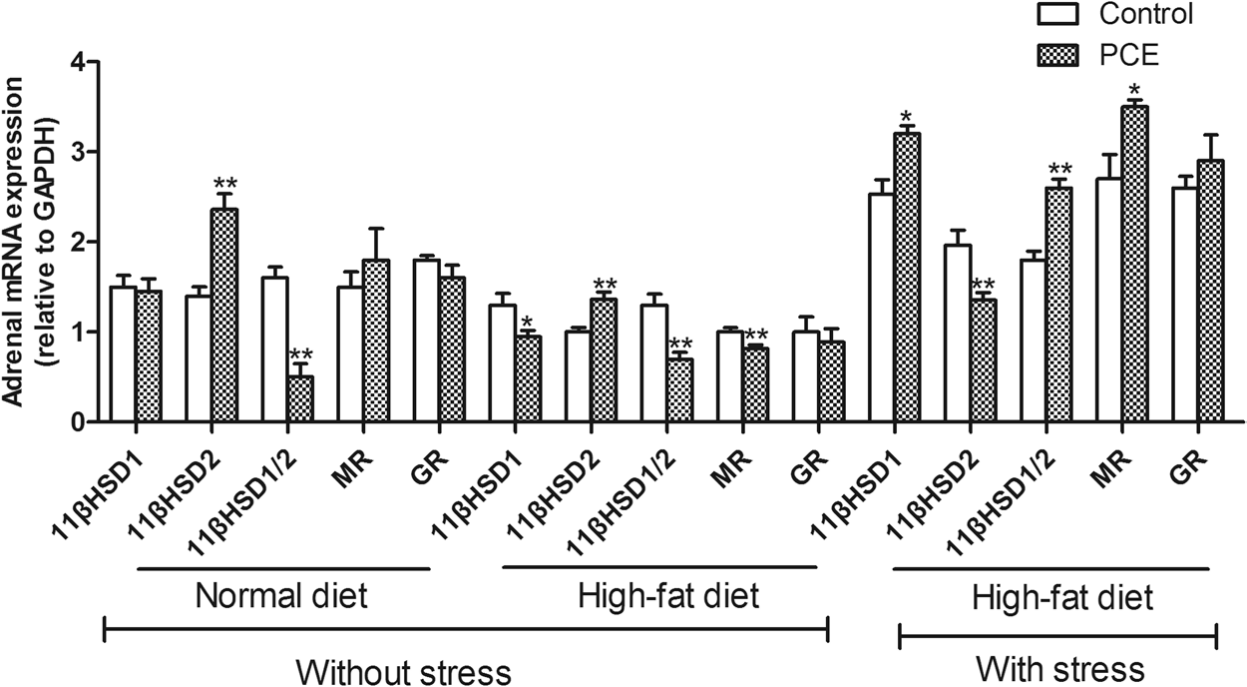
Effects of prenatal caffeine (PCE, 120 mg/kg.d) exposure on the mRNA expression of adrenal 11β-hydroxysteroid dehydrogenases (11βHSDs), glucocorticoid receptor (GR) and mineralocorticoid receptor (MR) with normal and high-fat diets without and with unpredictable chronic stress in adult male rat offspring. Mean ± S.E.M., n = 5, the adrenal gland samples from two litters were counted as one sample. ^*^ *P* < 0.05, ^**^ *P* < 0.01 *vs* respective control.

As shown in Fig. 4, compared with their respective controls, the PCE group fed a normal diet exhibited increased expression of 11βHSD2 (*P* < 0.01), which resulted in a decreased 11βHSD1/11βHSD2 ratio (*P* < 0.01), and the PCE group fed an HFD exhibited increased expression of 11βHSD2 (*P* < 0.01) as well as decreased expression of 11βHSD1, a decreased 11βHSD1/11βHSD2 expression ratio and decreased MR expression (*P* < 0.05, *P* < 0.01). With UCS exposure, the PCE group exhibited decreased expression of 11βHSD2 (*P* < 0.01) as well as increased expression of 11βHSD1, an increased 11βHSD1/11βHSD2 expression ratio and increased MR expression (*P* < 0.05, *P* < 0.01). These results demonstrated that the adrenal 11βHSD/CR system was suppressed under HFD conditions and activated with UCS conditions in male offspring with IUGR induced by PCE.

## Discussion

Developmental toxicity refers to permanent changes in morphology and function caused by damage in the early developmental stages^34^. The alteration of intrauterine programming is a permanent change in morphology and function caused by damage *in utero*, which is usually maintained from the developmental period into adulthood and even throughout the individual’s lifetime, leading to multiple effects on the adults^35^. Research has shown that high concentration of GC (dexamethasone) exposure during pregnancy can affect the sensitivity of the adrenal gland, mainly by increasing the mRNA expression of the ACTH receptor^36^. Our previous studies confirmed that PCE led to a low birthweight in fetal offspring rats and a high IUGR rate, and that offspring rats with PCE could undergo a period of catch-up growth after birth^11–13^. Furthermore, in the PCE-induced IUGR models, we found that the expression of StAR and CYP11A1 were reduced in the fetal adrenals due to overexposure to the maternal GCs^22^. Furthermore, when the PCE male offspring rats were fed a normal diet after birth, serum corticosterone levels presented a decreasing trend, the adrenal GC-activation system was inactivated, the expression of adrenal IGF1 signal pathway was enhanced, and the expression of the steroid synthetase system was increased. Hence, we had proposed a “two-programming” mechanism for PCE-induced adrenal developmental abnormality in the offspring rats, which included the low-function programming of adrenal *de novo* steroidogenesis and GC-IGF1 axis programming-mediated compensatory changes of steroidogenesis before and after birth^23^.

Studies have shown that an HFD can also affect the basic activity and stress sensitivity of the HPA axis^37,38^, and HFD exposure during pregnancy or lactation increased stress reactivity and serum corticosterone levels in spontaneously hypertensive adult rats^39^. Our previous study demonstrated that PCE induced a low expression of adrenal GC synthetase in the fetal rats and low basal activity of the HPA axis after birth^12,23^. Moreover, we had demonstrated an interaction between HFD after birth and PCE, which could affect the serum corticosterone levels^33^. In the present study, we found that the mRNA expression of the adrenal steroid synthetase system (including StAR, CYP11A1, 3β-HSD and CYP11B1) was reduced under HFD conditions. The immunohistochemical results showed that the protein levels of StAR and CYP11A1 were consistent with the gene expression, and the serum corticosterone concentration also showed a decreasing trend. These results suggested that the inhibited adrenal function in male offspring with PCE under HFD conditions may be the effect of programming, which was caused by the low basal activity of the HPA axis and the adrenal function inhibition. At the same time, the adrenal GC-activation system showed significant inactivation (reflected by effects such as decreased expression of 11β-HSD1 and increased expression of 11β-HSD2 as well as a decreased 11β-HSD1/11β-HSD2 ratio and decreased MR expression). Furthermore, the expression of the adrenal IGF1 signaling pathway components (including IGF1, IGF1R and AKT1) was notably increased, and the expression of the transcription factor for steroid synthetase, SF1, also showed an increasing trend. These results indicated that under HFD conditions, the inhibition of the GC-activation system may be related to the low serum GC level and may participate in the enhancement of the activity of the adrenal IGF1 signaling pathway (including SF1 expression) and steroid synthesis function. Hence, we hypothesized that the inactivation of GC-activation system increased adrenal steroidogenesis by enhancing the expression of the adrenal IGF1 signaling pathway, which then promoted local adrenal corticosterone synthesis and increased the serum corticosterone levels.

Studies have shown that chronic stress can affect the body’s homeostasis, such as by interfering with the synthesis and secretion of adrenal GCs^40^, and chronic stress has a long-term impact on the activity of the HPA axis^41^. Additionally, chronic stress can seriously affect the body’s endocrine and metabolic balance, increasing susceptibility to a variety of diseases, including cardiovascular disease and type 2 diabetes^42–44^. Our previous study found that ethanol exposure during pregnancy could change HPA axis activity in offspring under HFD and UCS conditions, which increased the susceptibility of the offspring to MS^45^. Caffeine exposure during pregnancy also could result in high stress sensitivity of the HPA axis in offspring rats after birth under normal diet conditions^11^. In the present study, we found that compared with the HFD control, the serum GC level and adrenal synthetase system expression were increased in the PCE male offspring with UCS, which suggested that UCS could improve adrenal steroid synthesis, leading to an increased serum GC level. Furthermore, the adrenal GC-activation system was triggered and the IGF1 signaling pathway was inhibited with UCS. These results showed that the UCS-induced high serum GC level could inhibit the adrenal IGF1 signaling pathway *via* negative feedback by activating the GC-activation system, which then decreased local corticosterone synthesis to maintain homeostasis.

Numerous experiments have shown that high blood GC levels increased the blood glucose level and increased the risk of insulin resistance, diabetes and other metabolic diseases^46–48^. Our previous additional study found that blood glucose and lipid levels and liver and pancreatic function were all damaged by PCE under normal diet conditions^34^. Meanwhile, an HFD could change HPA axis function in offspring rats with PCE, leading to a GC-associated blood glucose and lipid changes, which increased the susceptibility of the offspring to MS in a gender-specific manner^33^. In the present study, regardless of HFD or UCS conditions, the greater adrenal sensitivity of male offspring rats caused by PCE led to abnormal changes in adrenal synthesis, in which the GC-IGF1 axis played an important role by maintaining the stability of the serum GC level, serving a possible endocrine negative feedback axis. Furthermore, the serum GC level in the PCE group with HFD and UCS exposure was higher than in any other group (such as the normal diet group and the HFD group), which suggested that the compensatory effect maintained adrenal synthesis, and it could have easily caused the depletion and decompensation of adrenal function as well. Once decompensated, the glucose and lipid metabolic functions were affected, resulting in an enhanced susceptibility to MS and related metabolic diseases.

It is worth mentioning that gender differences in intrauterine programming of fetal development related research is commonly reported^32,33,36^. Meanwhile, another study indicated that prenatal corticosterone exposure induced offspring adrenal function abnormity in male but not female offspring^49^. Although only the male offspring rats were included in the present study, we tested the female rats at the same time. We were surprised to see some obvious gender differences in the GC-activation system and IGF1 signaling pathway of the PCE offspring rats under normal diet and HFD conditions, especially in the females, there was even disorder after UCS (data not shown). In view of the importance of gender differences in fetal origin adult diseases, we will strengthen the gender difference and its potential mechanism-associated research in the future.

One limitation of the present study is that the adrenal steroid synthetase system changes only were measured in gene expression not protein expression based on the limited amount of samples, but the immunohistochemistry of StAR and P450scc were included in the results. The other limitations are intragastric administration and litter size correction after birth. As a major confounding factor, caffeine administration by gavage is a stimulus and it can affect the corticosterone secretion of the pregnant rats, although the animals of the control group were given equal dose of saline also by gavage, which could relatively eliminate the gavage influence. Additionally, the litter size probably changed the offspring growth profile and metabolic health, therefore, we had corrected the litter size in both the experimental and control groups after birth, which would minimize the individual difference.

## Conclusions

PCE induced greater adrenal sensitivity in adult male offspring rats, as shown by the reduced corticosterone synthesis under HFD conditions and the significantly enhanced corticosterone levels with UCS exposure. Adrenal GC-IGF1 axis programming changes may play an important role in these alterations by maintaining the stability of the blood GC level *via* regulation of local corticosterone synthesis through negative feedback. The adaptive changes resulting from the GC-IGF1 axis programming may maintain blood GC level within a certain range. This study provides an experimental basis for clarifying PCE-caused adrenal dysfunction and abnormal circulating GC levels, which would be beneficial in addressing GC-associated diseases in the IUGR-affected offspring.

## Materials and Methods

### Chemicals and reagents

Caffeine (CAS #58-08-2, N99% purity) was purchased from Sigma-Aldrich Co., Ltd. (St. Louis, MO, USA). Isoflurane was purchased from Baxter Healthcare Co. (Deerfield, IL, USA). A rat corticosterone ELISA kit was obtained from Assay-pro LLC. (Saint Charles Missouri, USA). Reverse transcription and real-time quantitative PCR (RT-qPCR) kits were purchased from TaKaRa Biotechnology Co., Ltd. (Dalian, China). The oligonucleotide primers for rat genes were synthesized by Sangon Biotech Co., Ltd. (Shanghai, China). All other chemicals and agents were of analytical grade.

### Animals and treatment

Specific pathogen-free (SPF) Wistar rats (with weights of 200–240 g for females and 260–300 g for males) were obtained from the Experimental Center of Hubei Medical Scientific Academy (No. 2008–0005, Hubei, China). The animal experiments were performed at the Center for Animal Experiment of Wuhan University (Wuhan, China), which has been accredited by the Association for Assessment and Accreditation of Laboratory Animal Care International (AAALAC International). All animal experimental procedures were approved by and performed in accordance with the Guidelines for the Care and Use of the Animal Welfare Committee (AWC) of Wuhan University and the International Council on Research Animal Care; the AWC specifically oversees the university’s animal programs, equipment and procedures.

Animals were held under temperature-controlled conditions on a 12 h light: dark cycle and had *ad libitum* access to standard chow and tap water at all times. After one week of acclimation, two females were mated with one male for one night. Upon confirmation of mating by the appearance of sperm in a vaginal smear, the day was defined as gestational day (GD) 0. Pregnant females were then transferred to individual cages.

**Figure 5:**
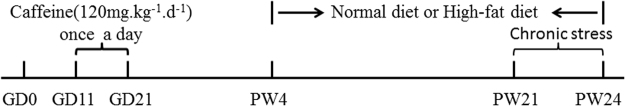
The schedule of animal treatment from gestation day (GD) 0 to postnatal week (PW) 24.

Pregnant rats were randomly divided into two groups: a control group and a PCE group. Ten dams were in each group. Starting from GD11 until term delivery (GD20), the PCE group was administered 120 mg/kg.d caffeine *via* oral gavage, as previously reported^12^. The control group was administered the same volume of vehicle. A schematic of the treatments for maternal and offspring rats is shown in Fig. 5. The pregnant rats were allowed to deliver spontaneously at term.

On postnatal day (PD) 1, the number of pups in each litter was set to 8 (♂:♀ = 1:1) randomly; to ensure adequate and standardized nutrition until weaning^50^. At postnatal week (PW) 4, 60 male pups from 20 different mothers (3 male pups were chosen from each mother) were selected randomly for each group (30 pups with IUGR from the caffeine group and 30 normal pups from the control group), and all pups were fed a normal diet or HFD *ad libitum* before being sacrificed. The standard rodent chow purchased from the Experimental Centre of Hubei Medical Scientific Academy contained 21% kcal from protein, 68.5% kcal from carbohydrate and 10.5% kcal from fat. The HFD was previously described by our laboratory^51^ and contained 88.0% corn flour, 11.5% lard, and 0.5% cholesterol, which provided 18.9% kcal from protein, 61.7% kcal from carbohydrate and 19.4% kcal from fat.

At PW-21, 40 male rats fed a normal diet or HFD were anesthetized with isoflurane and decapitated in a room separate from where the other animals were kept. To minimize the effect of corticosterone circadian rhythm, all the rats were sacrificed within 9:00 am to 11:00 am. Trunk blood was collected, and serum was prepared by centrifugation at 17,205 g for 15 min at 4 °C and stored at −80 °C until use for detection of the corticosterone concentration. The adrenal glands were dissected for RT-PCR and fixed in 4% formaldehyde for histological examination. The remaining 20 male rats fed an HFD were exposed to UCS for 3 weeks^52^, with the stress administered once daily between 8:30 am and 10:30 am with lights -on except for the stressor with 24 h duration. The stressors consisted of ① food deprivation for 24 h; ② water deprivation for 24 h; ③ tail pinch (2 cm from the end of the tail) for 5 min; ④ heating in an oven at 45 °C for 5 min; ⑤ cold swimming at 4 °C–8 °C for 4 min, after which the rats were towel dried; ⑥ reversed day-and-night cycles; and ⑦ social isolation (one rat per cage) for 24 h. Every stressor was administered randomly at an interval of 7 days, for a total of three times within 21 days. On the last day of stress (PW-24), after 12 h fasting, all rats were obliged to swim at 4 °C–8 °C for 4 min and were then towel -dried. One hour after swimming, the rats were anesthetized with isoflurane and decapitated in a room separate from where the other animals were kept. Trunk blood was collected, and serum was prepared by centrifugation at 17,205 g for 15 min at 4 °C and stored at −80 °C until use for detection of the corticosterone concentration. The adrenal glands were dissected for RT-qPCR and fixed in 4% formaldehyde for histological examination.

### Analysis of blood samples

The serum corticosterone concentration was detected using an ELISA kit following the manufacturer’s protocol. The limit of detection for the corticosterone concentration was 0.39 ng/mL. The intra-assay and inter-assay coefficients of variation for corticosterone were 5.0% and 7.2%, respectively. The cross-reactivity of the corticosterone ELISA, as defined by the manufacturer, was 2% for progesterone and 2% for aldosterone.

### RT-qPCR detection

Total RNA was isolated from the rat adrenal glands using TRIzol reagent according to the manufacturer’s protocol. The same tissues of littermates were pooled for homogenization. The concentration and purity of the isolated RNA were determined by spectrophotometry and adjusted to 1 μg/μl. Total RNA was stored in diethyl pyrocarbonate-H_2_O (DEPC-H_2_O) at −80 °C.

For RT-qPCR analysis, single-stranded cDNA was prepared from 2 μg of total RNA according to the protocol of the ExScript RT reagent kit. Primers were designed using Primer Premier 5.0, and their sequences are shown in Table 1. Relative standard curves were constructed for the target genes (Table 1), using the corresponding RT-qPCR products, isolated using a DNA extraction kit, at different concentrations, ranging from 10–10,000 pg per reaction. PCR was performed in 96-well optical reaction plates using the ABI Step One real-time PCR thermal cycler (ABI StepOne, NY, USA) in a 20 µl reaction mixture. To quantify the gene transcripts more precisely, the mRNA level of the housekeeping gene glyceraldehyde phosphate dehydrogenase (GAPDH) was measured and used as a quantitative control, with each sample normalized against GAPDH mRNA content. The PCR cycling conditions used were as follows: pre-denaturation, 95 °C for 30 s; denaturation, 95 °C for 5 s; annealing, the conditions for each gene are listed in Table 1; and elongation, 72 °C for 30 s (the elongation step was performed for the 3β-HSD reactions).

**Table 1:** Oligonucleotide primers and PCR conditions of rat in quantitative real-time PCR.

Genes	Forward primer	Reverse primer	Product (bp)	Annealing
StAR	GGGAGATGCCTGAGCAAAGC	GCTGGCGAACTCTATCTGGGT	188	65 °C, 30 s
CYP11A1	GCTGCCTGGGATGTGATTTTC	GATGTTGGCCTGGATGTTCTTG	188	63 °C, 30 s
CYP21	AGGAGCTGAAGAGGCACAAG	GAGGTAGCTGCATTCGGTTC	188	63 °C, 30 s
CYP11B1	CCCCTTTGTGGATGTGGTAG	CACGCTCTCAGGTTTCAGGT	188	61 °C, 30 s
3β-HSD	TCTACTGCAGCACAGTTGAC	ATACCCTTATTTTTGAGGGC	271	58 °C, 30 s
IGF1	GACCAAGGGGCTTTTACTTCAAC	TTTGTAGGCTTCAGCGGAGCAC	148	60 °C, 30 s
IGF1R	GTCCTTCGGGATGGTCTA	TGGCCTTGGGATACTACAC	188	62 °C, 30 s
Akt1	ATGAGCGACGTGGCTATTGTGAAG	GAGGCCGTCAGCCACAGTCTGGATG	156	60 °C, 30 s
SF1	CCAGTACGGCAAGGAAGA	GAGGCTGAAGAGGATGAGGA	188	63 °C, 30 s
11β-HSD1	GAAGAAGCATGGAGGTCAAC	GCAATCAGAGGTTGGGTCAT	133	63 °C, 30 s
11β-HSD2	TGGCCAACTTGCCTAGAGAG	TTCAGGAATTGCCCATGC	76	63 °C, 30 s
MR	TGCATGATCTCGTGAGTGA	GAGGCCGTCAGCCACAGTCTGGATG	156	63 °C, 30 s
GR	CACCCATGACCCTGTCAGTC	AAAGCCTCCCTCTGCTAACC	156	63 °C, 30 s
GAPDH	GCAAGTTCAATGGCACAG	AAGTTCTTCCTGGCCGGTAT	140	63 °C, 30 s

StAR: steroidogenic acute regulatory protein, CYP11A1: cytochrome P450 cholesterol side chain cleavage, 3β-HSD: 3β-hydroxysteroid dehydrogenase, CYP21: steroid 21-hydroxylase, CYP11B2: steroid 11β-hydroxylase, IGF-1: insulin-like growth factor-1, IGF-1R: IGF-1 receptor, AKT1: protein kinase B, SF1: steroidogenic factor 1, 11β-HSD1: 11β-hydroxysteroid dehydrogenase type 1, 11β-HSD2: 11β-hydroxysteroid dehydrogenase type 2, MR: mineralocorticoid receptor, GR: glucocorticoid receptor, GAPDH: glyceraldehyde 3-phosphate dehydrogenase.

### Immunohistochemical examination

The adrenal glands were fixed in 4% paraformaldehyde solution overnight and then processed using the paraffin-sectioning technique. The immunohistochemical procedures were performed using a streptavidin-peroxidase (SP)-conjugation method according to the manufacturer’s instructions. Paraffin-embedded tissues were cut into 5 μm-thick serial sections and stained for StAR and CYP11A1 with a goat monoclonal antibody (1:200 dilution; sc-7846, Santa Cruz Biotechnology). At least five random fields from each section were examined. The signal was visualized using light microscopy and imaged, and the positively stained areas were analyzed using the Photo Imaging System (Nikon H550S, Japan). The number of Ki67-stained nuclei in each image was also counted with the Photo Imaging System.

### Statistical analysis

SPSS 17 (SPSS Science Inc., Chicago, IL, USA) and Prism (GraphPad Software, La Jolla, CA, USA) were used for data analysis. All data presented are expressed as the mean ± S.E.M. The significant differences between different treatment groups were assessed with one-way analysis of variance (ANOVA). The gain rates of serum parameters were transformed if necessary before one-way ANOVA evaluations. Statistical significance was set at *P* < 0.05.
